# Supplementary material for: varVAMP: degenerate primer design for tiled full genome sequencing and qPCR
Source: Nat Commun. 2025 May 31;16:5067. doi: 10.1038/s41467-025-60175-9 (PMC12126543; doi:10.1038/s41467-025-60175-9)
Supplement: Supplementary file 1 — Supplementary Information [file 41467_2025_60175_MOESM1_ESM.pdf]

a

Alignment preprocessing

Example input alignment:

small gap　　　large gap

```
1  GAGCCCCTCTTGGAAGTCCATGGTGAGGGATTTGATACCTCACCGCCGTTTG---CCTAGGCTATAGGCTAAATTTCCCTT----------T
2  GAGCCCCTCTTGGAAGTCCATGGTGAGGGATTTGATACCTCACCGCCGTTTG---CCTAGGCTATAGGCTAAATTTCCCTT----------T
3  GAGCCCCTCTTGGAAGTCCATGGTGAGGGATTTGATACCTCTCCGCCGTTTG---CCTAGGCTATAGGCTAAATCTCCCTT----------T
4  GAGCCCCTCTTGGAAGCCCATGGTGAGGGATTTGATACCTCTCCGCCGTTTG---CCTAGGCTATAGGCTAAATTTCCCTT----------T
5  GACCCCCTGTTGGAAGCCCATGGTGAGGGAGTTGATACCTCGCCGCCGTTTG---CCTAGGCTATAGGCTAAATTTCCCTT----------T
6  GAACCCCTGTTGGTAGTCCATGGTGAGGGGACTTGATACCTCGCCGCCGTTTG---CCTAGGCTAAAGGCTAAATTTCCCTTTCGATTTCGAT
7  GAACCCCTGTTGGAAGTCCATGGTGAGGGGACTTGATACCTCCCCGCCGTTTG---CCTAGGCTAAAGGCTAAATTTCCCTTTCGATTTCGAT
8  GAACCCCTGTTGGAAGTCCATGGTGAGGGGACTTGATACCTCCCCGCCGTTTG---CCTAGGCTAAAGGCTAAATTTCCCTTTCGATTTCGAT
9  GAACCCCTCTTGGAAGTCCATGGTGAGGGGACTTGATACCTCACCGCCGTTTG---CCTAGGCTAAAGGCTAAATTTCCCTT----------T
10 GAACCCCTCTTGGAAGTCCATGGTGAG-GGACTTGATACCTCACCGCCGTTTGTTTCCTAGGCTATAGGCTAAATTTCCCTTTCGATTTCGAT
```

Preprocessed alignment (e.g. threshold 0.8):

```
1  gagcccctcttggaagtccatggtgaggggatttgatacctcaccgccgtttgNcctaggctataggctaaatttcccttNNt
2  gagcccctcttggaagtccatggtgaggggatttgatacctcaccgccgtttgNcctaggctataggctaaatttcccttNNt
3  gagcccctcttggaagtccatggtgaggggatttgatacctctccgccgtttgNcctaggctataggctaaatctcccttNNt
4  gagcccctcttggaagcccatggtgaggggatttgatacctctccgccgtttgNcctaggctataggctaaatttcccttNNt
5  gacccctgttggaagcccatggtgaggggagttgatacctcgccgccgtttgNcctaggctataggctaaatttcccttNNt
6  gaacccctgttggtagtccatggtgaggggacttgatacctcgccgccgtttgNcctaggctaaaggctaaatttcccttNNt
7  gaacccctgttggaagtccatggtgaggggacttgatacctccccgccgtttgNcctaggctaaaggctaaatttcccttNNt
8  gaacccctgttggaagtccatggtgaggggacttgatacctccccgccgtttgNcctaggctaaaggctaaatttcccttNNt
9  gaacccctcttggaagtccatggtgaggggacttgatacctcaccgccgtttgNcctaggctaaaggctaaatttcccttNNt
10 gaacccctcttggaagtccatggtgag-ggacttgatacctcaccgccgtttgNcctaggctataggctaaatttcccttNNt
```

b

Consensus creation e.g. threshold 0.8

Nucleotide counts of columns with differences:

a:	5	0	9	0	0	0	4	4	0
t:	0	0	1	8	0	4	2	5	9
c:	1	6	0	2	0	5	2	1	1
g:	4	4	0	0	9	1	2	0	0
-:	0	0	0	0	1	0	0	0	0

Characters to reach consensus threshold e.g. 0.8:

ag　cg　a　tc　g　ct　atcg　ta　t

ambiguous consensus: garcccctsttggaagyccatggtgaggggayttgatacctcnccgccgtttgNcctaggctawaggctaaatttcccttNNt

majority consensus: gaacccctcttggaagtccatggtgaggggacttgatacctcaccgccgtttgNcctaggctataggctaaatttcccttNNt

c

Primer region search (e.g. 2 allowed ambiguous characters within 18 nucleotides)

(16 chars)　(51 chars)　(26 chars)

ambiguous consensus: garcccctsttggaagyccatggtgaggggayttgatacctcnccgccgtttgNcctaggctawaggctaaatttcccttNNt

region_1 cccctcttggaagtccatggtgaggggacttgatacctcaccgccgtttg

region_2 cctaggctataggctaaatttccctt

d

K-mer digestion (minimum primer length - maximum primer length)

region_2 (51 chars) cccctcttggaagtccatggtgaggggacttgatacctcaccgccgtttg

```
cccctcttggaagtcca     gaggggacttgatacct
cccctcttggaagtccat    gaggggacttgatacctc
cccctcttggaagtccatg   gaggggacttgatacctca
 …                     …
 ccctcttggaagtccat
 ccctcttggaagtccatg
 ccctcttggaagtccatgg
 …
  cctcttggaagtccatg
  cctcttggaagtccatgg
  cctcttggaagtccatggt
  …
```

Supplementary Figure 1. (continued on the next page)

e

Primer evaluation

Each k-mer is hardfiltered if certain constraints (user definable) are not met:

- Not in temperature range
- Not in GC range
- Over base penalty
- Over maximum homodimer temperature threshold
- Over maximum hairpin temperature threshold
- Over/under minimum GC content at 3’ end
- Presence of ambiguous characters within the last x bases of 3’ end
- Over maximum allowed polyX characters
- Over maximum allowed dinucleotide repeats

Each passing primer is given its penalty:
base penalty + permutation penalty + 3’ mismatch penalty

Primer penalties (user-definable multipliers)

Base penalty:
Diff. to opt length * length_multiplier +
Diff. to opt gc * gc_multiplier +
Diff. to opt temp * temp_multiplier

Permutation penalty:
number of primer permutations * multiplier

3’ penalty:
Frequency of mismatches at 3’ position *
3’ position multiplier

f

Select best primers

Sort primers by penalty (low to high) -> define overlapping primers with the middle third of a retained primer

Example:

ggcggggtcaactccatgattag	Primer1: penalty 0.7
ggcggggtcaactccatgatt	Primer2: penalty 1.7
atggcggggtcaactccatgatt	Primer3: penalty 0.8

Primer1 is retained, Primer2 and Primer3 are excluded

g

Find amplicons

Find all amplicons within amplicon size constraints. Example:

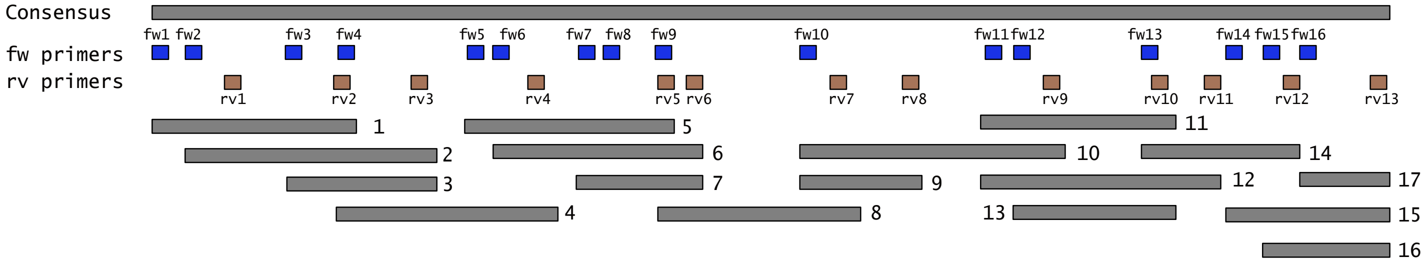

amplicon_penalty = (penalty fw primer + penalty rv primer) * e^(amplicon length/optimal length)

h

Create graph

Transform amplicons into a graph. Amplicons are only connected if the start of B overlaps with the second half of A and is not within in the minimum overlap region of A.

Example how two connected nodes are created:

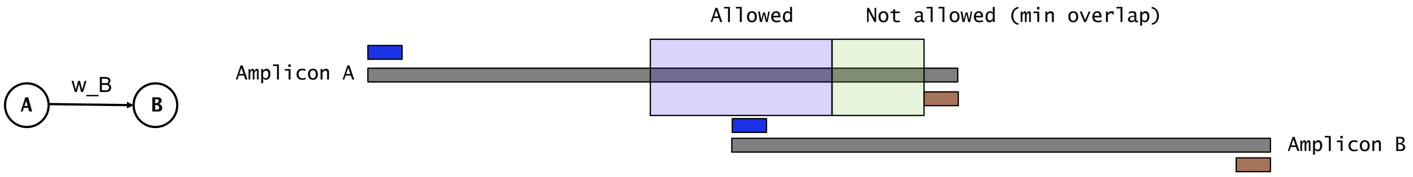

Edge weight w_B of A->B: (off-target hits of B -> True/False (1/0), penalty of B)

Supplementary Figure 1. (continued on the next page)

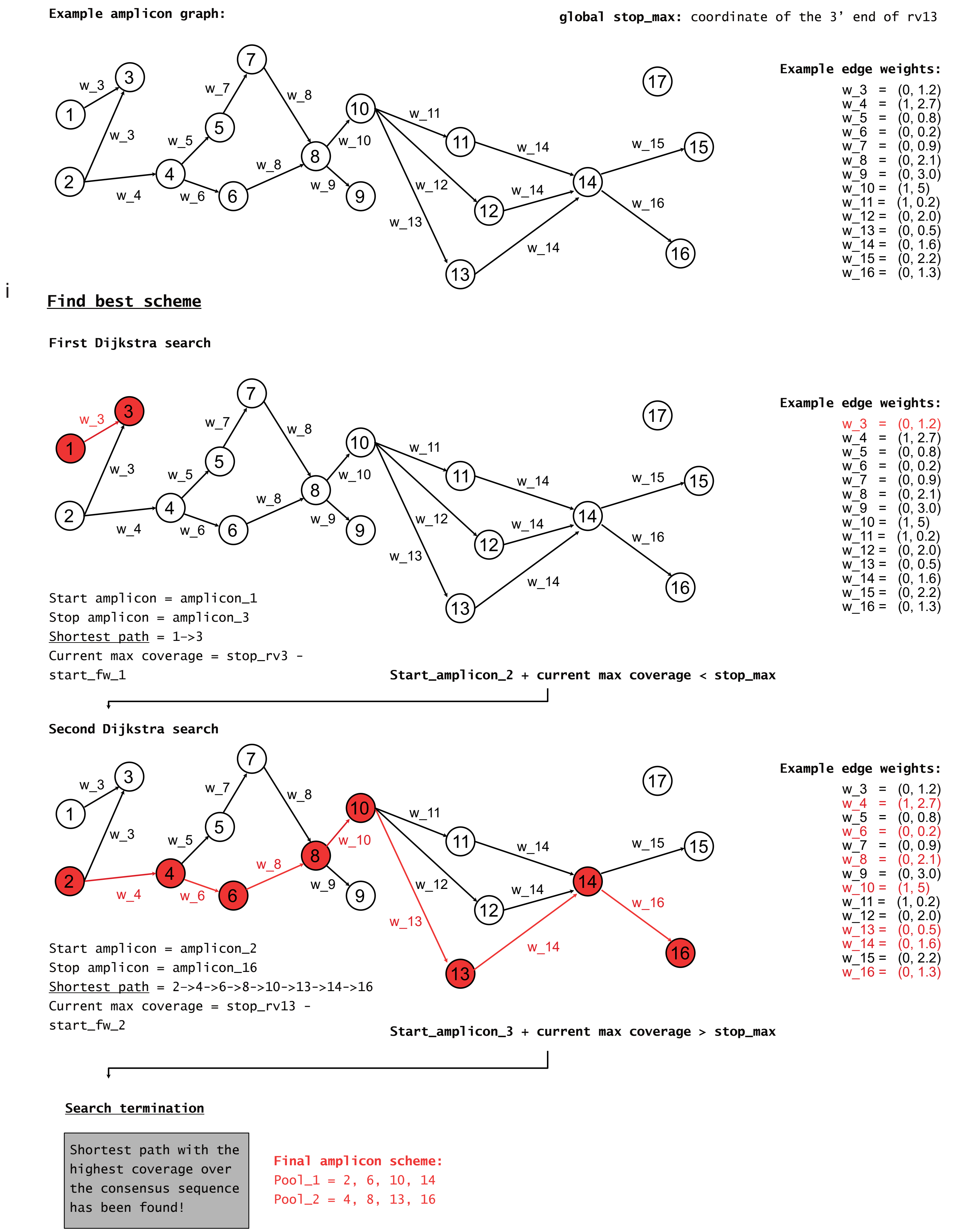

Supplementary Figure 1 Detailed varVAMP workflow. The main algorithmic steps of varVAMP are illustrated in examples. (a) The alignment is first pre-processed by lower character conversion and gap-cleaning and then (b) two consensus sequences are created based on the threshold parameter. (c) Next, primer regions are searched, (d) digested into k-mers, (e) and then tested for a variety of primer parameters and penalized. (f) Afterwards, primers are subsampled based on their penalty and (g) all potential amplicons within the length constraints are computed. (h) Finally, the amplicons are converted to an amplicon graph and (i) searched with Dijkstra's algorithm to create the final amplicon scheme.

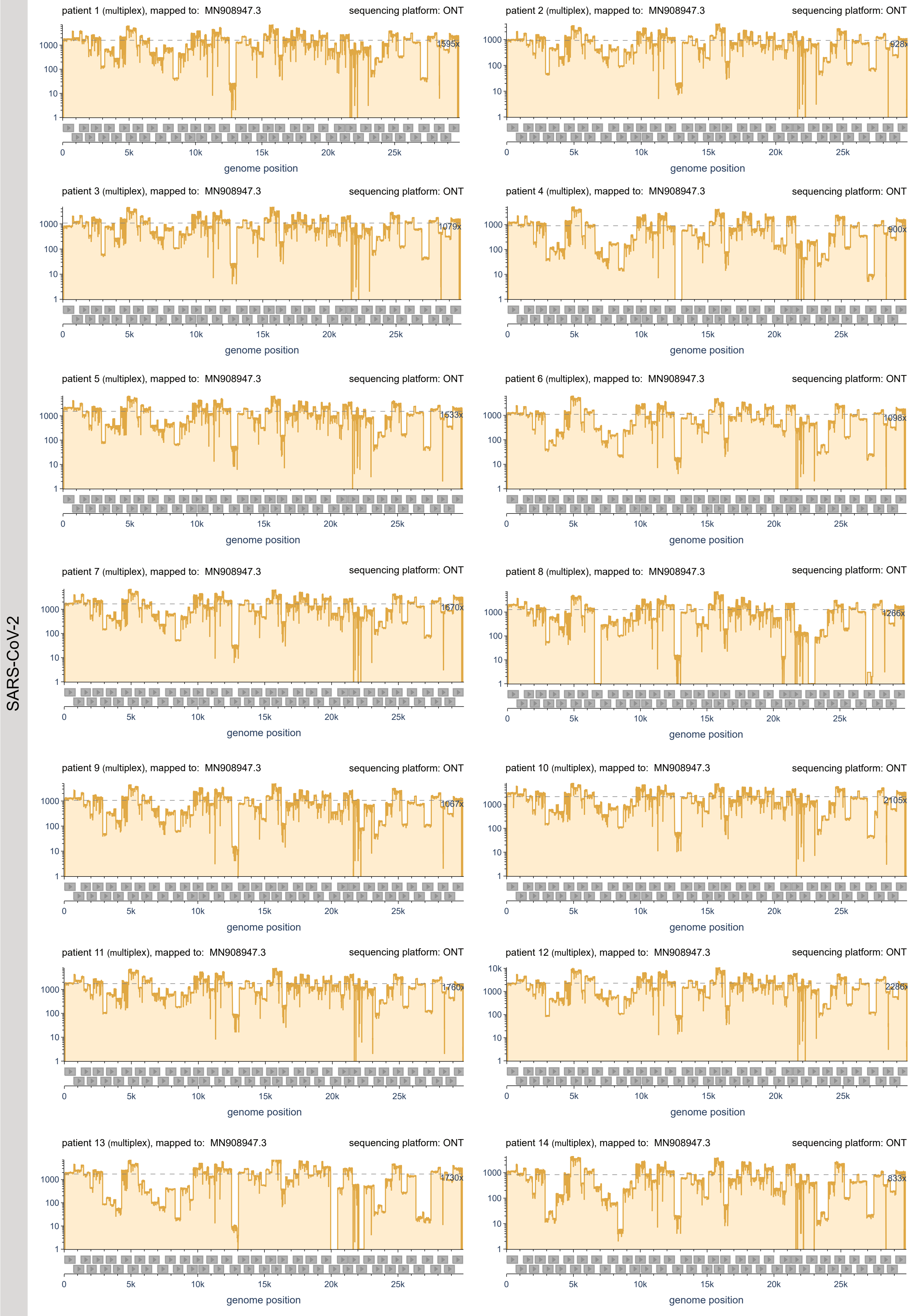

Supplementary Figure 2. (continued on the next page)

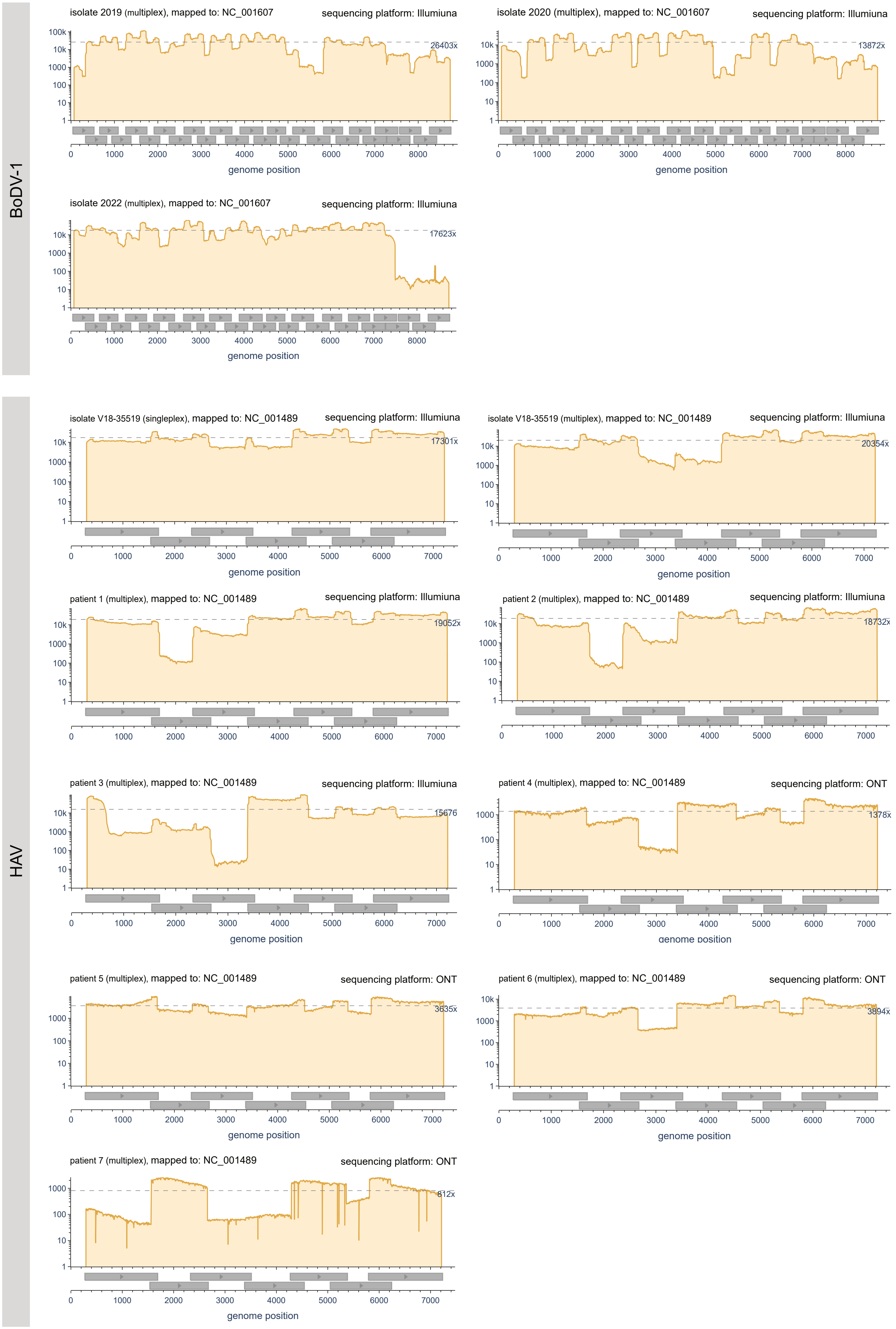

Supplementary Figure 2. (continued on the next page)

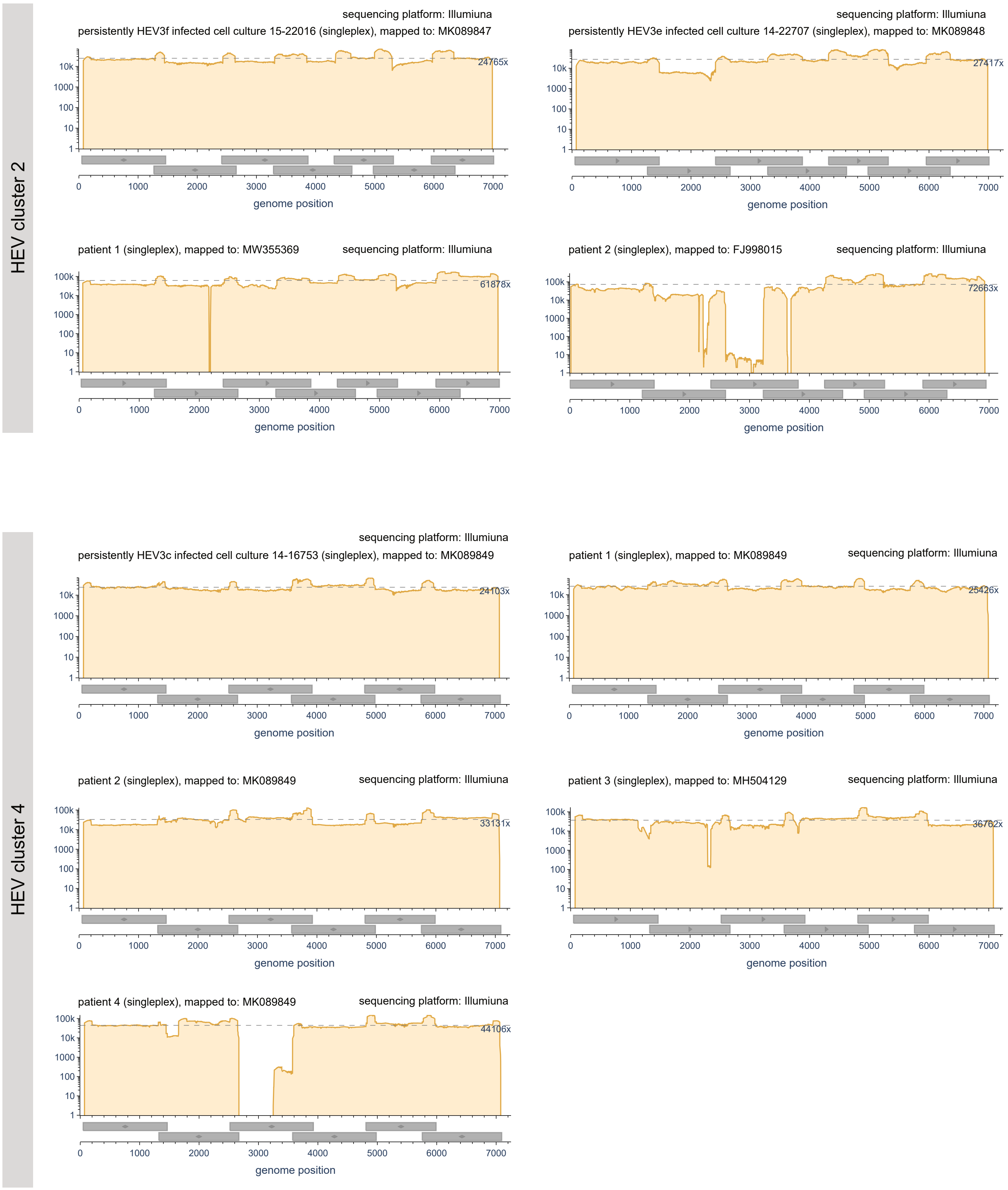

Supplementary Figure 2. (continued on the next page)

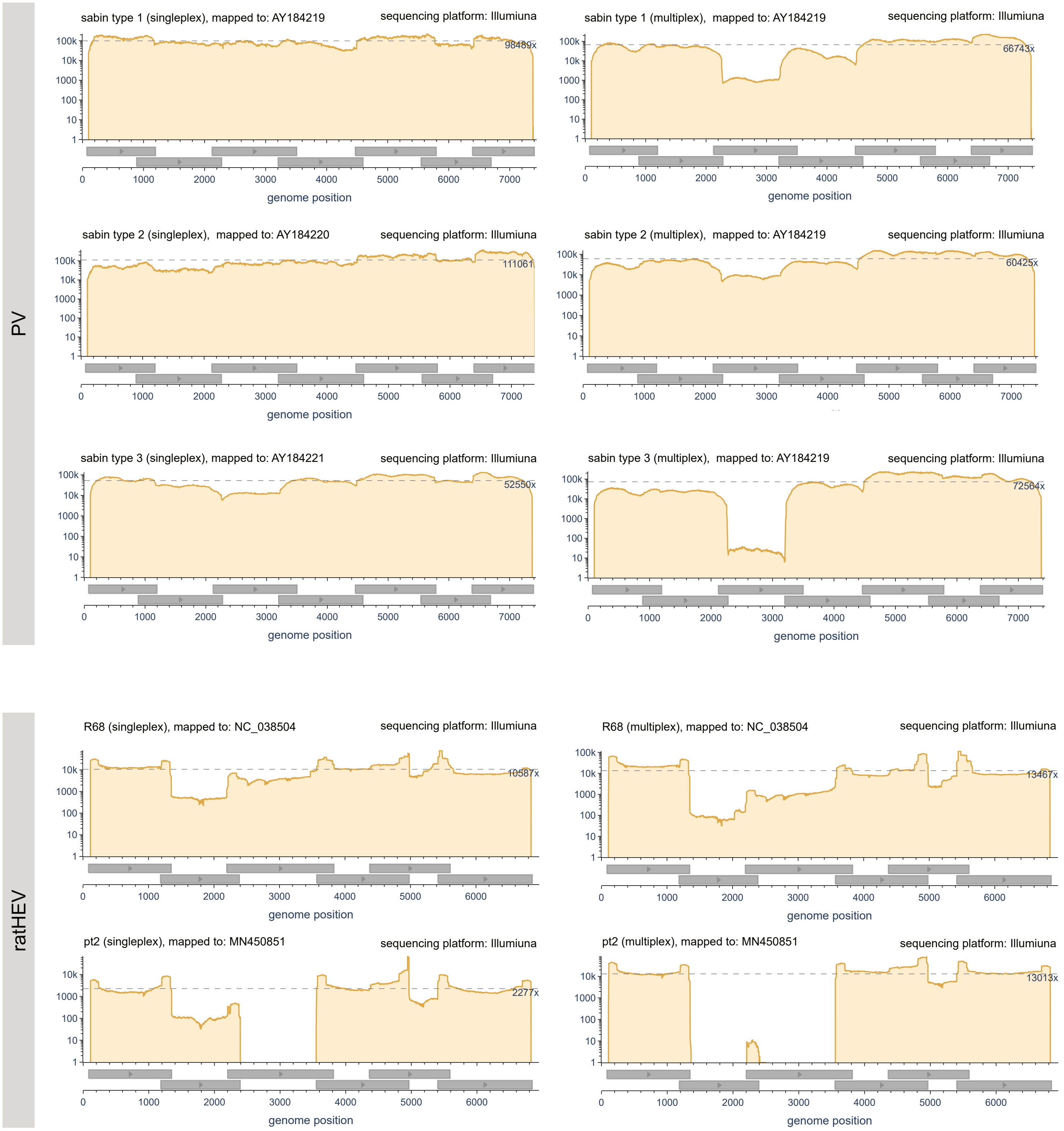

Supplementary Figure 2. Coverage plots for all sequencing results. Coverage plots of the different SARS-CoV-2, BoDV-1, HAV, PV and ratHEV samples subjected to their respective tiled amplicon whole genome Illumina sequencing workflows. The dotted lines indicate the mean coverages. NCBI accession numbers of the reference sequences used for mapping are indicated in the headers. Coverage plots were created with BAMdash.

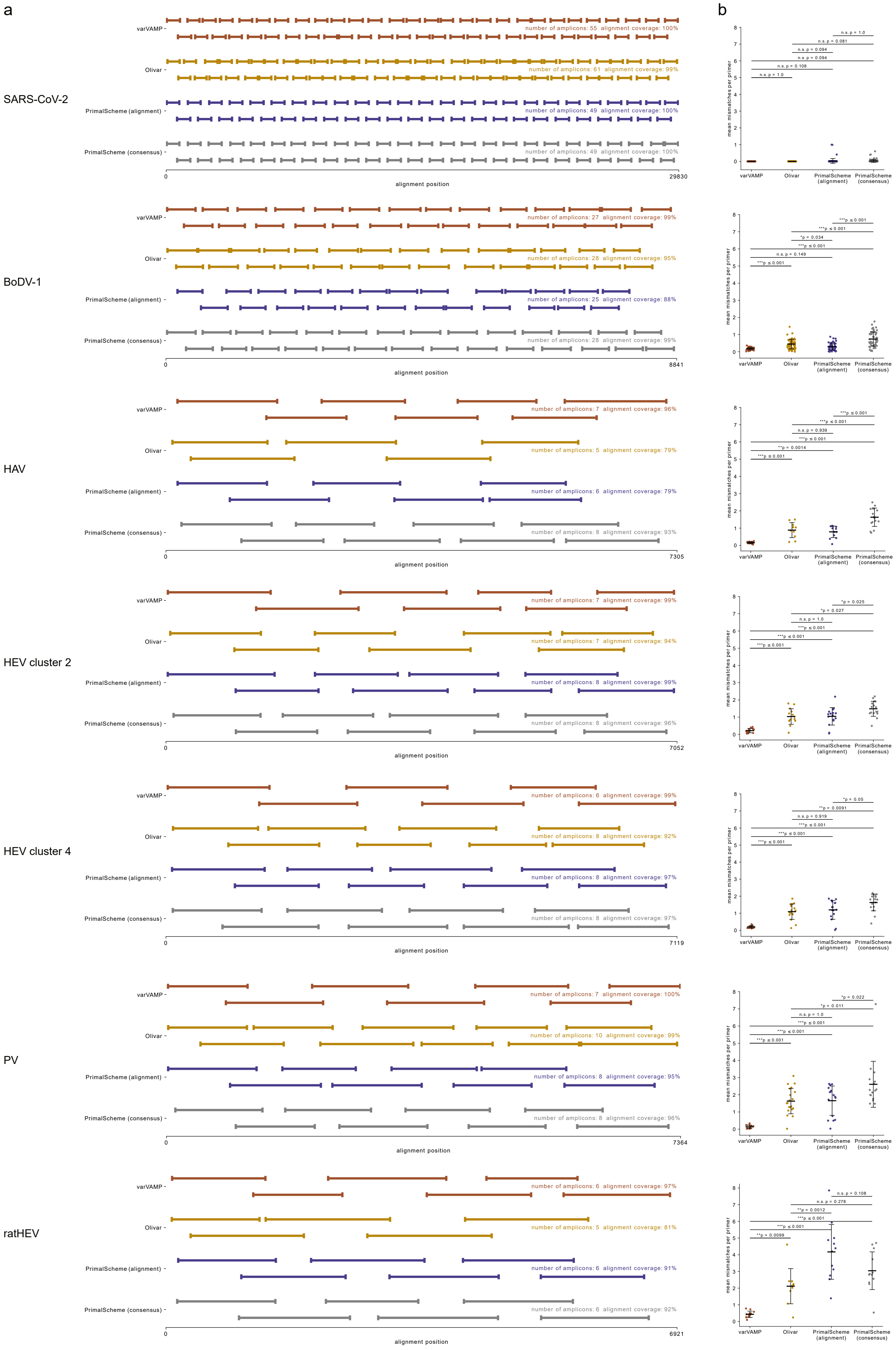

Supplementary Figure 3. Overview of the varVAMP, Olivar and PrimalScheme primer schemes and comparison of the mean mismatches. (a) Overview of the different primer schemes designed with varVAMP, Olivar, PrimalScheme on the basis of a subsampled MSA and PrimalScheme on the basis of a consensus sequence. Amplicon positions were deduced from the respective bed files. **(b)** Mean mismatches per primer with the alignment sequence. Each dot represents the mean mismatch of a single primer. The means and standard deviations within the respective schemes are given. Statistics were calculated using a one-way ANOVA and Tukey's multi-comparison post-hoc test (n.s. - not significant, *: $p \leq 0.05$, **: $p \leq 0.01$, ***: $p \leq 0.001$).

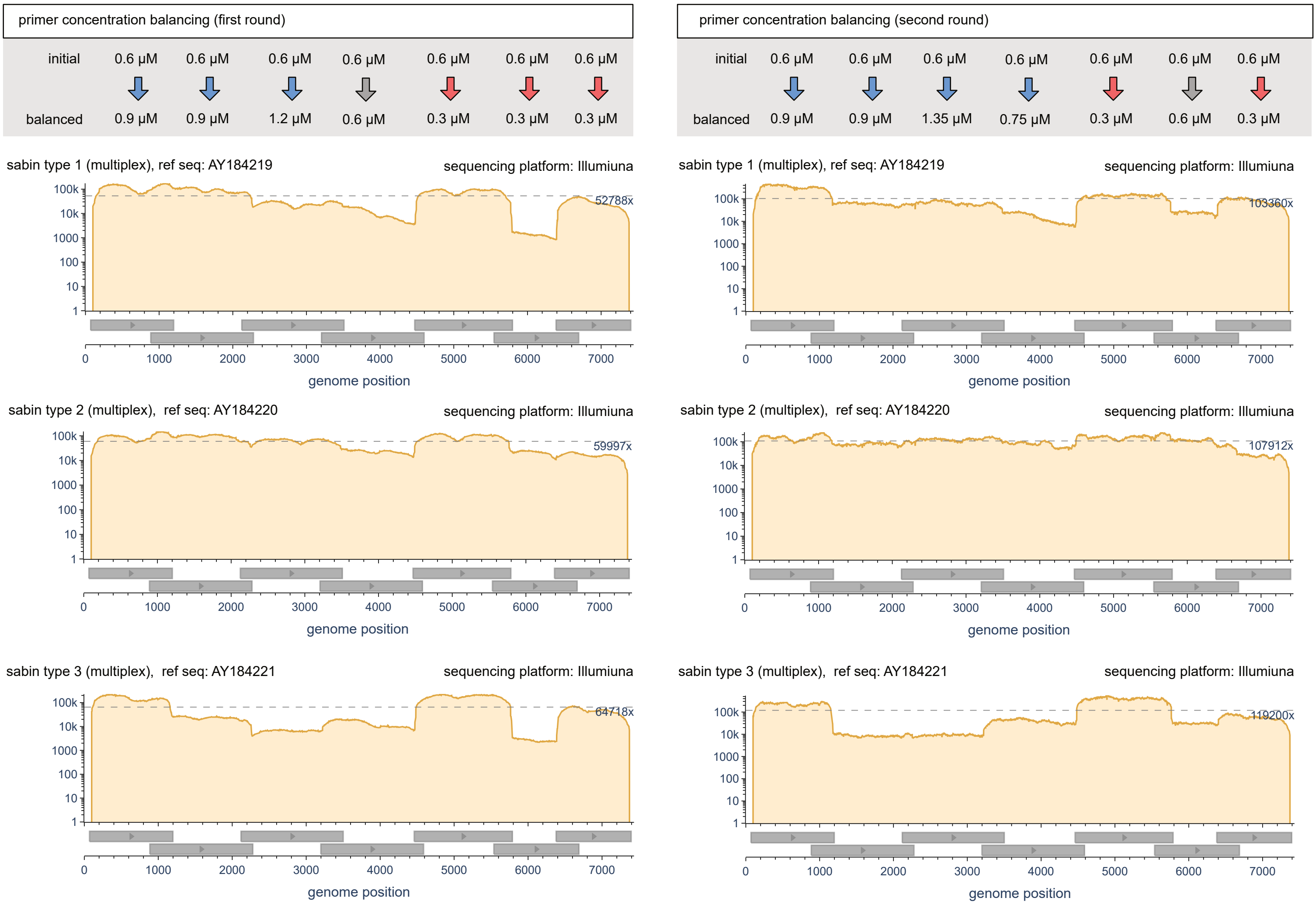

Supplementary Figure 4. Primer balancing for PV whole genome sequencing. The PV tiled primers initially used at equimolar concentrations for multiplex reactions were balanced in two consecutive rounds on the basis of prior results and then the balanced primers were used in multiplex PCRs for Sabin 1-3 prior Illumina sequencing. The respective concentrations for each iteration are given above the coverage plots (blue arrow - increase in molarity, gray arrow - no change in molarity, red arrow - decrease in molarity). The dotted lines indicate mean coverages. NCBI accession numbers of the reference sequences used for mapping are indicated in the headers. Coverage plots were created with BAMdash.

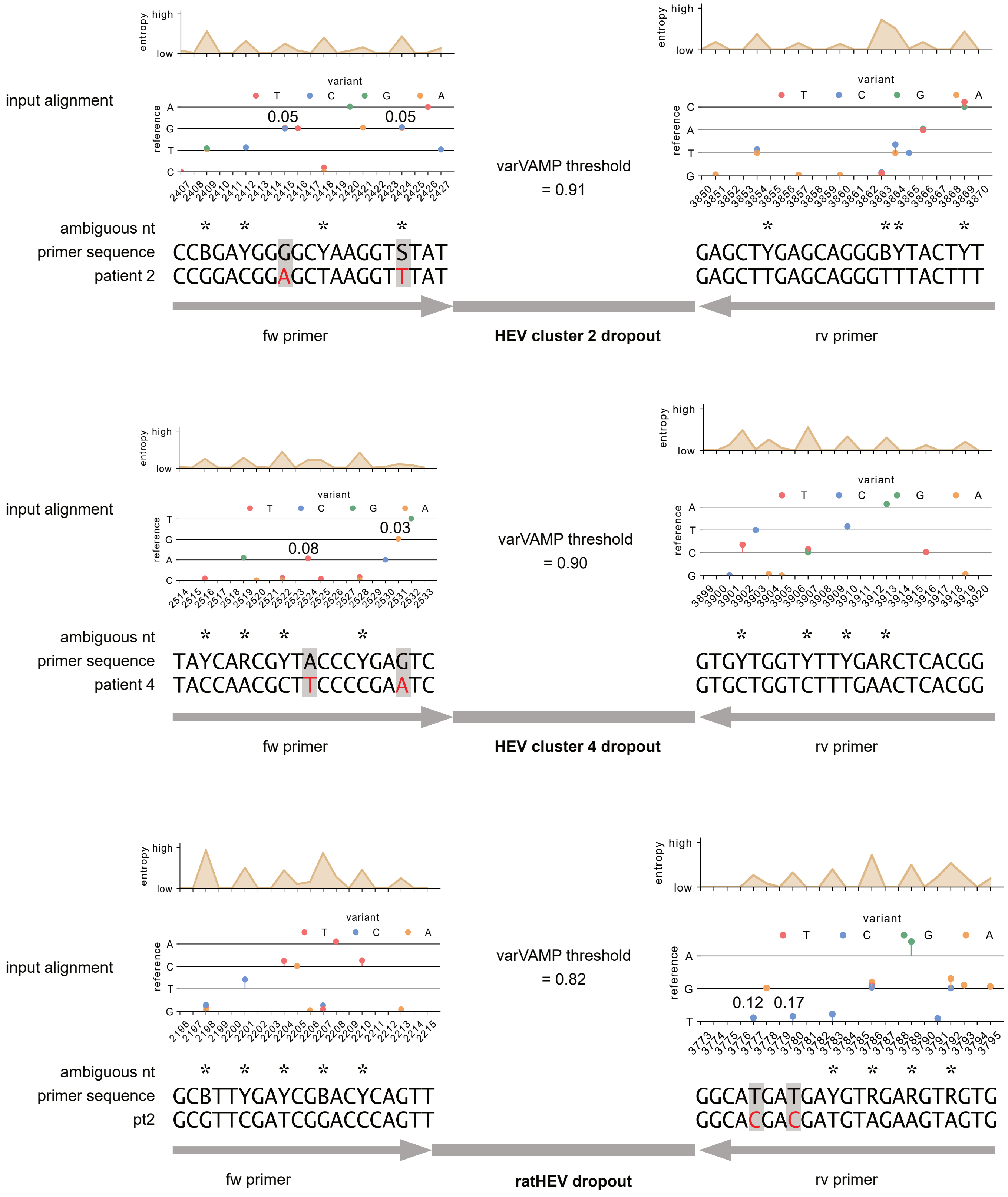

Supplementary Figure 5. Amplicon dropout analysis. For all amplicon dropouts, the target sequence was compared with the primer sequence and mismatches highlighted in red. Moreover, the respective regions in the input alignments were analysed with MSAexplorer v.0.1 (https://github.com/jonas-fuchs/MSAexplorer) showing both the entropy and observed variants compared to the alignments consensus sequence. All mismatches were found in the input alignment and their frequency is indicated above the respective dots

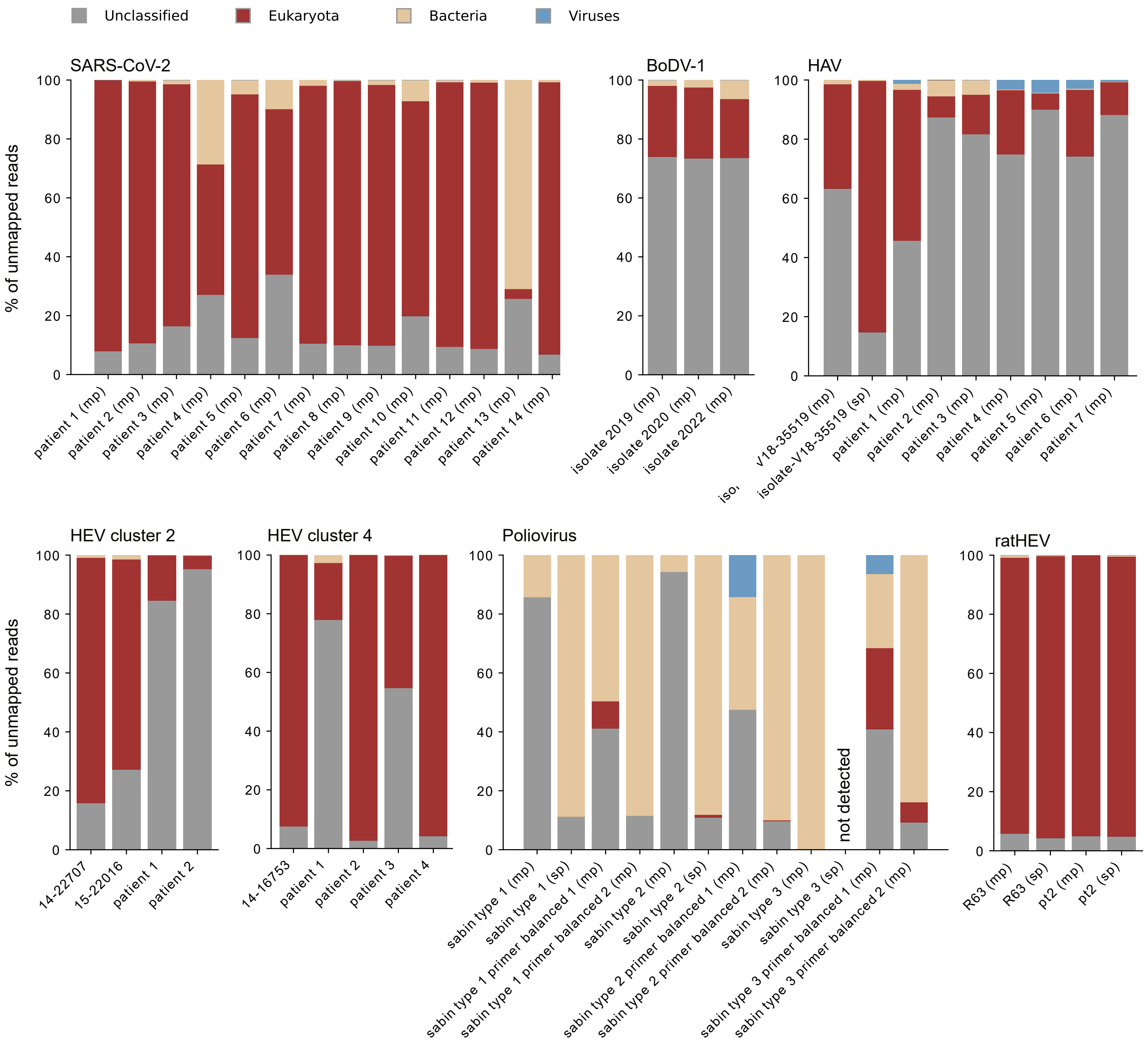

Supplementary Figure 6. Off-target amplifications for each amplicon scheme and sample. For each sample, unmapped reads were extracted and analysed with Kraken2 using the Minikraken v2 database. Shown are the main kingdoms where reads were assigned to. Notably, the Minikraken v2 database only contains one eukaryotic reference (human).
