# Supplementary material for: varVAMP: degenerate primer design for tiled full genome sequencing and qPCR
Source: Nat Commun. 2025 May 31;16:5067. doi: 10.1038/s41467-025-60175-9 (PMC12126543; doi:10.1038/s41467-025-60175-9)
Supplement: Supplementary file 2 — Reporting summary [file 41467_2025_60175_MOESM2_ESM.pdf]

nature portfolio

Corresponding author(s): Jonas Fuchs

Last updated by author(s): May 6, 2025

Reporting Summary

Nature Portfolio wishes to improve the reproducibility of the work that we publish. This form provides structure for consistency and transparency in reporting. For further information on Nature Portfolio policies, see our Editorial Policies and the Editorial Policy Checklist.

Statistics

For all statistical analyses, confirm that the following items are present in the figure legend, table legend, main text, or Methods section.

n/a	Confirmed	
☒	☐	The exact sample size (*n*) for each experimental group/condition, given as a discrete number and unit of measurement
☐	☒	A statement on whether measurements were taken from distinct samples or whether the same sample was measured repeatedly
☐	☒	The statistical test(s) used AND whether they are one- or two-sided<br>*Only common tests should be described solely by name; describe more complex techniques in the Methods section.*
☒	☐	A description of all covariates tested
☒	☐	A description of any assumptions or corrections, such as tests of normality and adjustment for multiple comparisons
☐	☒	A full description of the statistical parameters including central tendency (e.g. means) or other basic estimates (e.g. regression coefficient) AND variation (e.g. standard deviation) or associated estimates of uncertainty (e.g. confidence intervals)
☐	☒	For null hypothesis testing, the test statistic (e.g. *F*, *t*, *r*) with confidence intervals, effect sizes, degrees of freedom and *P* value noted<br>*Give P values as exact values whenever suitable.*
☒	☐	For Bayesian analysis, information on the choice of priors and Markov chain Monte Carlo settings
☒	☐	For hierarchical and complex designs, identification of the appropriate level for tests and full reporting of outcomes
☒	☐	Estimates of effect sizes (e.g. Cohen's *d*, Pearson's *r*), indicating how they were calculated

Our web collection on statistics for biologists contains articles on many of the points above.

Software and code

Policy information about availability of computer code

Data collection	no software was used for data collection
Data analysis	Primer design with varVAMP:<br>varVAMP (different versions - indicated in table 1) - currently latest version: v.1.2.2 (DOI: 10.5281/zenodo.14826629)<br>covsonar v.1.1.9<br>sc2-mutation-frequency-calculator v.0.0.2<br>fasta36 (ggsearch) v.36.3.8<br>vsearch v.2.22.1<br>IQ-TREE v.2.0.7<br>MAFFT v.7.490<br>identity v.2.0<br><br>Primerdesign with Olivar:<br>SNP-sites v.2.5.1<br>Olivar v1.1.5<br><br>Primerdesign with PrimalScheme:<br>PrimalScheme v.1.4.1<br>FastTree2 v.2.1.11<br>PARNAS v.0.1.5

ONT sequencing:
MinKNOW software version 23.07.12

NGS analysis:
Galaxy 24.1.2
fastp v0.20.1
BWA-MEM v.0.7.17
ivar trim v1.3.1
LoFreq v2.1.5
BCFtools v1.15.1
poreCov v1.9.3
minimap2 v2.17
medaka v1.8.0
Dorado v.0.5.3
vcflib Version 1.0
samtools v.1.9
bedtools v.2.30.0
freebayes v.1.3.6

Off-targets
BEDtools v2.27.1
Kraken2 v.2.1.3

qPCR analysis
Roche LightCycler 480 II device software version LCS480 1.5.1.62

Data visualization:
BAMdash v.0.2.4
https://github.com/jonas-fuchs/varVAMP_in_silico_analysis v.1.1
GraphPad Prism 8

For manuscripts utilizing custom algorithms or software that are central to the research but not yet described in published literature, software must be made available to editors and reviewers. We strongly encourage code deposition in a community repository (e.g. GitHub). See the Nature Portfolio guidelines for submitting code & software for further information.

Data

Policy information about availability of data

All manuscripts must include a data availability statement. This statement should provide the following information, where applicable:

- Accession codes, unique identifiers, or web links for publicly available datasets
- A description of any restrictions on data availability
- For clinical datasets or third party data, please ensure that the statement adheres to our policy

Genome recovery raw data (Figure 2 and 5) and qPCR data is provided with this paper (source data file).

Remaning raw data and scripts to reproduce the in silico analysis and software benchmark have been deposited at: https://github.com/jonas-fuchs/varVAMP_in_silico_analysis v.1.2 (DOI: 10.5281/zenodo.14826645)

Raw sequencing data was deposited at ENA under the accession number: PRJEB74744

All input multiple sequence alignments and varVAMP outputs for primers that have been evaluated in this study are available at: https://github.com/jonas-fuchs/ViralPrimerSchemes (DOI: 10.5281/zenodo.10562883)

Research involving human participants, their data, or biological material

Policy information about studies with human participants or human data. See also policy information about sex, gender (identity/presentation), and sexual orientation and race, ethnicity and racism.

Reporting on sex and gender	Gender or sex was not analysed in this study.
Reporting on race, ethnicity, or other socially relevant groupings	Race, ethnicity, or other socially relevant groupings were not analysed in this study.
Population characteristics	No population characteristics were analysed in this study.
Recruitment	Patients were not specifically recruited. Their samples were choosen based on virus positive qPCR results during diagnostic routine.
Ethics oversight	The ethical oversight depended on the respective centers:<br>- Next generation sequencing was performed in the Diagnostic Department of the Institute of Virology, University Medical Center, Freiburg using pseudonymized patient left over specimens from HEV positive patients (Local ethics committee no. 1001913).

- For HAV patient samples, the molecular diagnostics were collected and analysed on the basis of the German Infection Protection Act. Thus, a review by an ethics committee was not required.
- Analyses of SARS-CoV-2 positive samples collected within the IMSSC2 network were conducted according to the principles laid down in the Helsinki Declaration with anonymized data as described (Oh et al., Clin Infect Dis. 2022; 75(S1): S110-20).

Note that full information on the approval of the study protocol must also be provided in the manuscript.

Field-specific reporting

Please select the one below that is the best fit for your research. If you are not sure, read the appropriate sections before making your selection.

☒ Life sciences ☐ Behavioural & social sciences ☐ Ecological, evolutionary & environmental sciences

For a reference copy of the document with all sections, see nature.com/documents/nr-reporting-summary-flat.pdf

Life sciences study design

All studies must disclose on these points even when the disclosure is negative.

Sample size	Sample sizes were predetermined due to the limited available number of diagnostic samples or virus isolates for the respective primer schemes.
Data exclusions	For the primer design with PrimalScheme, we had to limit the number of sequences in the initial alignment to under 200 due to software restrictions . Therefore the alignments with >200 sequences were subsampled for phylogenetic representative sequences. Moreover, a few sequences that were too short had to also be excluded due to restrictions by PrimalScheme (detailed in the material and methods section).
Replication	Each sample (patient sample or virus isolate) was sequenced once and in isolated cases twice (single- and multiplex reactions). The reproducibility of the respective primer schemes was shown by sequencing multiple samples for each scheme.
Randomization	No randomization was applied as no group allocation was carried out.
Blinding	Blinding was not applied as all available patient material/data were used and therefore blinding did not affect the experiments and analyes. Non-objective parameters were not included in the study design.

Reporting for specific materials, systems and methods

We require information from authors about some types of materials, experimental systems and methods used in many studies. Here, indicate whether each material, system or method listed is relevant to your study. If you are not sure if a list item applies to your research, read the appropriate section before selecting a response.

Materials & experimental systems

n/a	Involved in the study	
☒	☐	Antibodies
☐	☒	Eukaryotic cell lines
☒	☐	Palaeontology and archaeology
☒	☐	Animals and other organisms
☒	☐	Clinical data
☒	☐	Dual use research of concern
☒	☐	Plants

Methods

n/a	Involved in the study	
☒	☐	ChIP-seq
☒	☐	Flow cytometry
☒	☐	MRI-based neuroimaging

Eukaryotic cell lines

Policy information about cell lines and Sex and Gender in Research

Cell line source(s)	Vero cells (ATCC: CCL-81)<br>PLC/PRF/5 (ATCC: CRL-8024)<br>HuH-7-Lunet BLR cells<br>HuH-7<br>RD-A
Authentication	None of the cell lines were authenticated.
Mycoplasma contamination	all cell lines were tested monthly negative for mycoplasma
Commonly misidentified lines<br>(See ICLAC register)	no commonly misidentified cell lines were used in the study

Plants

Seed stocks	*Report on the source of all seed stocks or other plant material used. If applicable, state the seed stock centre and catalogue number. If plant specimens were collected from the field, describe the collection location, date and sampling procedures.*
Novel plant genotypes	*Describe the methods by which all novel plant genotypes were produced. This includes those generated by transgenic approaches, gene editing, chemical/radiation-based mutagenesis and hybridization. For transgenic lines, describe the transformation method, the number of independent lines analyzed and the generation upon which experiments were performed. For gene-edited lines, describe the editor used, the endogenous sequence targeted for editing, the targeting guide RNA sequence (if applicable) and how the editor was applied.*
Authentication	*Describe any authentication procedures for each seed stock used or novel genotype generated. Describe any experiments used to assess the effect of a mutation and, where applicable, how potential secondary effects (e.g. second site T-DNA insertions, mosiacism, off-target gene editing) were examined.*
